# Research on Interest Rate Risk of Housing Mortgage Loan Based on Computer Simulation

**DOI:** 10.1155/2021/6035022

**Published:** 2021-08-23

**Authors:** Enlin Tang

**Affiliations:** ^1^Graduate School, University of Perpetual Help System DALTA, Manila 1740, Philippines; ^2^School of Finance and Mathematics, Huainan Normal University, Huainan, Anhui 232038, China

## Abstract

In recent years, with the rapid increase of the business volume of housing mortgage loans of commercial banks, the risk of prepayment is increasingly exposed. Prepayment will have a great impact on the duration and convexity of housing mortgage loans of commercial banks and then bring difficulties to the asset liability management of banks. Therefore, empirical research on the changes of duration and convexity of housing mortgage loans caused by prepayment when the market interest rate changes is of great significance for commercial banks to manage interest rate risk exposure. Based on the analysis of the option characteristics of prepayable housing mortgage loan, the CIR model with GARCH(1, 1) is selected to describe the interest rate change path, and the computer simulation method is used to calculate OAS and then calculate the effective duration and effective convexity of housing mortgage loan under different prepayment rates, so as to understand the interest rate risk of housing mortgage loan in the presence of embedded option.

## 1. Introduction

Since the 1980s, China's real estate market began to start. After several years of tortuous development, it entered a stable development stage in 1995. Since then, China's real estate market has seized the important opportunity of urban housing system reform and started to take off. In the context of the prosperity and development of the real estate market, in 1992, China Construction Bank began to issue the first housing mortgage loan business. This innovative financial way enabled most people who could not afford to buy a house with full payment to realize their dream of buying a house. It not only greatly improved the consumption ability of Chinese urban residents but also stimulated the demand of the whole real economy. It promotes the rapid growth of GDP. When the total amount of personal consumption credit in the whole society is growing rapidly and the housing mortgage loan business of commercial banks is booming, the risks contained in the housing mortgage loan, as the main assets of banks, are increasingly exposed, especially the phenomenon of loan prepayment caused by the changes of benchmark interest rate of deposit loan and personal income is becoming more and more obvious. That is to say, the prepayment risk of housing mortgage is exposed [1]. In 2004, in the principles of interest rate risk management and supervision, the basic committee has clearly pointed out that the option risk caused by prepayment belongs to interest rate risk [2]. Under the background of interest rate marketization in China, commercial banks will face frequent and large fluctuation of benchmark interest rate of deposit and loan in the future, so commercial banks have to face up to the phenomenon of prepayment of housing mortgage loan and take active measures to deal with the interest rate risk of housing mortgage loan caused by prepayment [3]. Therefore, studying the impact of prepayment on the duration and convexity of housing mortgage loan from the microperspective can provide some ideas for commercial banks to manage interest rate risk.

## 2. Analysis on the Option Characteristics of Housing Mortgage Loan

### 2.1. Influencing Factors of Prepayment of Housing Mortgage Loan

#### 2.1.1. Interest Rate

The interest rate factor is the primary factor to determine the prepayment behavior. The interest rate factor here refers to the difference between the contract interest rate and the market interest rate. The contract interest rate determines the interest expense of the loan, and the market interest rate determines the refinancing cost for prepayment. Suppose the borrower prepays at time *t*, the cost of refinancing is expressed as *V*(*t*), the penalty is expressed as *α*, and the loan balance at *t* is expressed as *L*(*t*). When *W*(*t*)=*V*(*t*) − [*L*(*t*)+*α*] is greater than zero, the borrower will choose to prepay.

#### 2.1.2. Economic Factors

With the increase of population and family income under the three child policies, people's demand for improved housing increases, so there will be the phenomenon of prepaying the mortgage loan of old housing. In addition, when the national macroeconomy is running well, the whole real estate market is hot. When the rise of house prices exceeds the balance of mortgage loans, there will be a large number of prepayment behaviors.

#### 2.1.3. Institutional Factors

Commercial banks have formulated a series of regulations on prepayment of housing mortgage loans, such as the minimum repayment amount of each prepayment, the level of default interest for prepayment, and the limit of the times of prepayment.

#### 2.1.4. Other Factors

Other factors are relocation, characteristics of mortgage loans, and seasons [4–6].

### 2.2. An Analysis of Option Characteristics of Housing Loan

In order to clarify the option characteristics of prepayable housing mortgage loan more clearly, this paper assumes that a borrower and a commercial bank sign a loan contract with a term of *T* at time 0. In the contract, the loan amount is expressed as 1, the penalty is *α*, and the loan interest rate is expressed as continuous compound interest rate *r*_0,*T*_. Without considering prepayment, the total cost of borrowing is *e*^*r*_0,*T*_*T*^, assuming that the benchmark interest rate changes to *r*_1,*T*_ at time *t*, and the refinancing cost is (*e*^*r*_0,*T*_*t*^+*α*)*e*^*r*_1,*T*_(*T* − *t*)^; if *e*^*r*_0,*T*_*T*^ ≥ (*e*^*r*_0,*T*_*t*^+*α*)*e*^*r*_1,*T*_(*T* − *t*)^, the rational borrower will prepay; otherwise, the prepayment will not be executed. This method can be used for similar analysis at any time of interest rate adjustment.

At time *t*, the mathematical expression of the borrower's borrowing cost is

(1) $$\begin{array}{c} -\min\left[\left(\left(e^{r_{0,T}t}+\alpha \right)e^{r_{1,T}\left(T-t\right)}\right),e^{r_{0,T}T}\right]\\ =-e^{r_{0,T}T}-\min\left[\left(e^{r_{0,T}t}+\alpha \right)e^{r_{1,T}\left(T-t\right)}-e^{r_{0,T}T},0\right]\\ =-e^{r_{0,T}T}+\max\left[e^{r_{0,T}T}-\left(e^{r_{0,T}t}+\alpha \right)e^{r_{1,T}\left(T-t\right)},0\right]. \end{array}$$

In formula (1), −*e*^*r*_0,*T*_*T*^ represents a loan contract with default risk, and max[*e*^*r*_0,*T*_*T*^ − (*e*^*r*_0,*T*_*t*^+*α*)*e*^*r*_1,*T*_(*T* − *t*)^, 0] represents an interest rate call option with the price of *K*=*e*^*r*_0,*T*_*T*^ implied in the loan contract.

**Figure 1:**
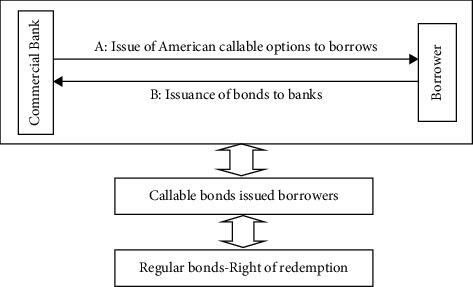
Decomposition of housing mortgage business.

The housing mortgage loan of commercial banks can be understood as the borrower issues bonds to the bank that can be repaid in advance. This kind of bonds implies options. At any time during the loan contract period, the borrower can choose to repay in advance. Therefore, such a loan contract can be decomposed into the borrower issuing callable bonds to the bank; the bank issued an American callable option to the borrower (as shown in Figure 1), in which A was often ignored by the bank, and the bank did not charge the option fee to the borrower [7, 8].

### 2.3. Measurement of Interest Rate Risk of Housing Mortgage Loan in the Presence of Embedded Options

When the embedded option exists, the traditional duration and convexity will lose the accuracy in measuring the interest rate risk of housing mortgage loan. At this time, in order to measure the interest rate risk of housing mortgage loan more accurately, we introduce effective duration *D*_eff_ and effective convexity *C*_eff_ [9], whose expressions are as follows:

(2) $$\begin{array}{c} D_{\text{eff}}=\frac{P_{+}-P_{-}}{2\Delta rP_{0}}, \end{array}$$

(3) $$\begin{array}{c} C_{\text{eff}}=\frac{P_{+}-2P_{0}+P_{-}}{\Delta r^{2}P_{0}}. \end{array}$$

The meanings of *P*_+_ and *P*_−_ in formulas (2) and (3) are as follows:

(4) $$\begin{array}{c} P_{+}=\frac{1}{m}\overset{m}{\underset{j=1}{\sum }}\overset{n}{\underset{t=1}{\sum }}\frac{{\text{CF}}_{t}^{j}}{\prod_{i=1}^{t}\left(1+r_{i}^{j}+\Delta r+\text{OAS}\right)}, \end{array}$$

(5) $$\begin{array}{c} P_{-}=\frac{1}{m}\overset{m}{\underset{j=1}{\sum }}\overset{n}{\underset{t=1}{\sum }}\frac{{\text{CF}}_{t}^{j}}{\prod_{i=1}^{t}\left(1+r_{i}^{j}-\Delta r+\text{OAS}\right)}. \end{array}$$

In formulas (2) and (3), Δ*r* is the base point of interest rate difference; *P*_0_ is the initial value of the bond; *P*_+_ represents the bond price when the interest rate moves upward by a certain base point; *P*_−_ is the bond price when the interest rate drops to a certain base point; and *m* in expressions *P*_+_ and *P*_−_ represents the number of interest rate change paths.

In formulas (4) and (5), *t* is the period of cash flow generation; *j* is the *j*-th path of interest rate change; *n* is the total term of the contract; CF_*t*_^*j*^ refers to the cash flow of period *t* under the path of interest rate change in article *j*; *r*_*i*_^*j*^ refers to the market interest rate of period *i* under the path of interest rate change in article *j*; and OAS is the risk compensation after eliminating the embedded option when the theoretical value of the bond is equal to the market value. OAS is the basis for calculating the effective duration and effective convexity; its mathematical expression is as follows:

(6) $$\begin{array}{c} P=\frac{1}{m}\sum_{j=1}^{m}\sum_{t=1}^{n}\frac{{\text{CF}}_{t}^{j}}{\prod_{i=1}^{t}\left(1+r_{i}^{j}+\text{OAS}\right)}, \end{array}$$

where *P* represents the market price of the bond; (1/*m*) is the mean value of risk neutral probability measure. In the formula, the calculation of OAS depends on the simulation of interest rate path. When simulating the interest rate path, the selected interest rate model should not only conform to the assumed stochastic process characteristics but also conform to the initial interest rate term structure. Then, based on cash flow and discount factor, *P*_+_ and *P*_−_ are obtained, and then the effective duration and effective convexity are calculated [10, 11].

## 3. Analysis on the Option Characteristics of Housing Mortgage Loan

### 3.1. Selection of Cases and Determination of Interest Rate Model

The 5-year housing loan of China Construction Bank with the amount of 300000 yuan is selected for analysis. The loan is repaid with equal principal and interest, and the starting date of the loan is November 1, 2019. At the same time, assuming that there are no extra expenses such as intermediate and tax, any amount of prepayment can be made, and the penalty is one month's repayment. For the sake of simplicity, the variables in the case are represented by letter parameters, as shown in Table 1.

In order to get the term structure of risk-free interest rate, we select the data of interest bearing bonds on November 1, 2019, to split the coupon from the interest bearing bonds. Using EViews software regression, the final expression of discount factor is as follows [12, 13]:

(7) $$\begin{array}{c} d\left(t\right)=a_{0}+a_{1}t+a_{2}t^{2}+a_{3}t^{3}=1-0.018121t-0.002416t^{2}+0.000099t^{3}. \end{array}$$

**Figure 2:**
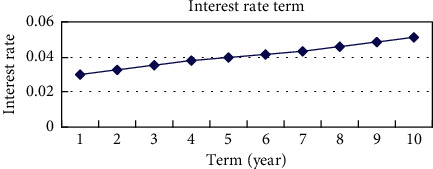
Risk-free interest rate term structure.

The risk-free interest rate data are further calculated by the discount factor and depicted, as shown in Figure 2.

As for the choice of interest rate model, the 7-day interbank offered rate from November 4, 2018, to November 2, 2020, is compounded, and then the ADF test is carried out. The results are shown in Table 2.

It can be seen from the results in Table 2 that the data do not have unit root and are a stationary series. Therefore, in the selection of interest rate model, we consider the CIR model, that is, it has the feature of mean reversion. However, it is found that the residual graph has the “clustering” feature during the fitting, and the ARCH − LM test with the lag order *P*=3 is further taken. The results are shown in Table 3.

It can be seen from Table 3 that the values of P are small, which means that the residual series has ARCH effect. Therefore, GARCH(1,1) is added to modify and the parameter estimation results are brought into the formula. The final expression of interest rate model is as follows:

(8) $$\begin{array}{c} \text{d}r=0.00989\left(0.96033-r\right)\text{d}t+1.00898\sigma_{\varepsilon }\sqrt{r}\text{d}z. \end{array}$$

In equation (8), *σ*_*ε*_ satisfies *σ*_*ε*_^2^=0.929687+0.348012*ε*_*t*−1_^2^+0.640113*σ*_*ε*_*t*−1__^2^, and then ARCH − LM test is carried out. The results are shown in Table 4.

The two *P* values in Table 4 are large, which indicates that the model with GARCH(1,1) can be used as an interest rate model.

### 3.2. Selection of Computer Simulation Methods

Monte Carlo simulation was developed in the mid-1940s to adapt to the development of atomic energy at that time. It is a random simulation method, also known as random sampling or statistical test method. Based on probability theory and mathematical statistics, Monte Carlo simulation belongs to a branch of computational mathematics. Traditional empirical methods cannot approach the real physical process, and it is difficult to get satisfactory results. Monte Carlo method can simulate the actual physical process, so it can get the results in line with the actual situation. The use of Monte Carlo simulation is very dependent on computer technology. The core of the problem is to convert the problem to probability model and to solve the problem by using random number or pseudorandom number. Therefore, it is necessary to realize random sampling by computer [14, 15]. In this article, in the process of calculating OAS, effective duration, and effective convexity, it is necessary to simulate the interest rate change path in advance. Therefore, Monte Carlo computer simulation method is used to describe the interest rate change path.

### 3.3. Empirical Analysis Based on Monte Carlo Simulation

Based on the above CIR interest rate model with GARCH(1,1), Monte Carlo simulation is carried out here. The loan cash flow is calculated on the basis of the simulated interest rate change path. The prepayment rate refers to the PSA prepayment rate model (as shown in formula (9)) [6]:

(9) $$\begin{array}{c} \text{CPR}=\left\{\begin{array}{cc} 0.2\%\cdot t, & 1\leq t\leq 30,\\ 6\%, & t>30. \end{array}\right) \end{array}$$

The calculation process of cash flow in phase 1 is as follows:

(10) $$\begin{array}{c} {\text{MB}}_{0}=300000,\\ {\text{MP}}_{1}={\text{MB}}_{0}\left[\frac{i{\left(1+i\right)}^{n-t+1}}{{\left(1+i\right)}^{n-t+1}-1}\right]=30\cdot \frac{i{\left(1+i\right)}^{60}}{{\left(1+i\right)}^{60}-1},\\ I_{1}={\text{MB}}_{0}*i=0.1323,\\ {\text{SP}}_{1}={\text{MP}}_{1}-I_{1}=0.4375,\\ {\text{PR}}_{1}={\text{SMM}}_{1}\left({\text{MB}}_{0}-{\text{SP}}_{1}\right)=0.05913,\\ C_{1}=I_{1}+{\text{SP}}_{1}+{\text{PR}}_{1}=0.62893,\\ C_{k}=I_{k}+{\text{SP}}_{k}+{\text{PR}}_{k},\ldots . \end{array}$$

**Figure 3:**
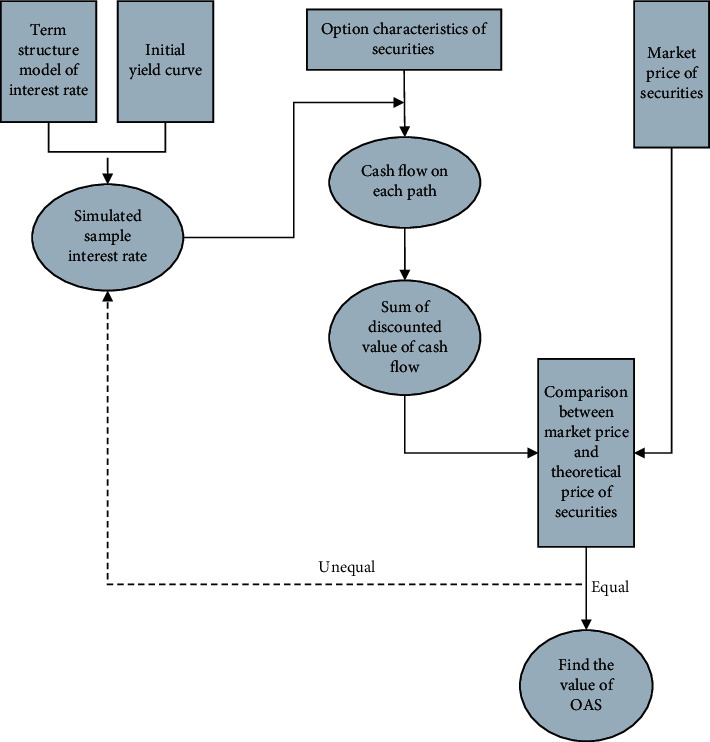
Calculation flow chart of option-adjusted spread.

Using the same principle and method, we calculate the cash flow of the following periods in turn, then discount the cash flow of each period, choose the sum of risk-free interest rate and OAS as the discount rate, and constantly try and error OAS until the weighted average of each cash flow is equal to the market price of mortgage assets [16–18]. The calculation flow chart of OAS is shown in Figure 3.

On the basis of OAS value, formulas (2) and (3) are used to calculate the effective duration and effective convexity, and the final calculation results are obtained, as shown in Table 5.

From the empirical results, when there is no prepayment (no embedded option) of the mortgage, the effective duration is 2.49863. When the prepayment rate is 50% PSA, the effective duration is 1.88. When the prepayment rate is 100% PSA, the effective duration is 1.6012, and the effective duration is 1.3952 when the prepayment rate is 150% PSA.

When there is no prepayment (no embedded option), the effective convexity is 138.8735; when the prepayment rate is 50% PSA, the effective convexity is 53.7065; when the prepayment rate is 100% PSA, the effective convexity is 6.6954; when the prepayment rate is 150% PSA, the effective duration is −118.9674.

## 4. Conclusion

1. When the embedded option exists, that is, the mortgage loan is allowed to prepay, the characteristics of duration and convexity of mortgage assets will change. The traditional duration and convexity will lose accuracy in measuring the interest rate risk of the mortgage loan, so it is necessary to introduce the effective duration and effective convexity to measure the interest rate risk.
2. The effective duration decreases with the increase of prepayment rate, and the effective convexity also decreases with the increase of prepayment rate. Especially when the prepayment rate reaches a certain degree, the effective convexity will become negative, that is, the value interest rate curve of assets bends in the opposite direction.
3. When the fluctuation of interest rate causes the borrowers of housing mortgage to choose prepayment, the interest rate risk of loan assets of commercial banks will appear. In this case, when managing the interest rate risk, commercial banks need to rematch the assets and liabilities according to the effective duration and effective convexity, so as to reduce or eliminate the interest rate risk exposure.

## Figures and Tables

**Table 1:** Parameters and significance.

Parameter	Significance
MP_*t*_	Repayment of phase *t*
MB_*t*−1_	Loan balance of phase *t* − 1
*i*_*b*_	7-day interbank offered rate
*i*=*i*_*b*_+2%	Mortgage interest rate
*n*	Total loan term
SMM_*t*_	Prepayment rate of phase *t*
MP_*t*_=MB_*t*−1_[*i*(1+*i*)^*n*−*t*+1^/((1+*i*)^*n*−*t*+1^ − 1)]	Repayment of phase *t*
*I*_*t*_=MB_*t*−1_*∗i*	Interest payable of phase *t*
SP_*t*_=MP_*t*_ − *I*_*t*_	Principal repayment amount of phase *t*
PR_*t*_=SMM_*t*_(MB_*t*_ − SP_*t*_)	Prepayment amount of phase *t*
MB_*t*+1_=MB_*t*_ − PR_*t*_ − SP_*t*_	Outstanding part of *t* + 1 principal
*C*_*t*_=*I*_*t*_+SP_*t*_+PR_*t*_	Cash flow from loans of phase *t*

**Table 2:** ADF test results.

ADF test statistic	−3.659969	1% critical value	−3.4013
		5% critical value	−2.8602
		10% critical value	−2.5698
MacKinnon critical values for rejection of hypothesis of a unit root			

**Table 3:** ARCH-LM test results.

Breusch-Godfrey serial correlation LM test			
F-statistic	2.200313	Prob. F (3,744)	0.0839
Obs*∗R* − squared	6.601124	Prob. chi-square (3)	0.0841

**Table 4:** ARCH-LM test results.

Heteroskedasticity test: ARCH			
*F* − statistic	0.279036	Prob. F (3,742)	0.8772
Obs*∗R* − squared	0.735012	Prob. chi − square (3)	0.8766

**Table 5:** Calculation results of effective duration and effective convexity.

	50% PSA		100% PSA		150% PSA		No embedded option	
Simulation results	2.161%	30.0995	2.24%	30.0502	2.31%	30.0462	1.91%	30.0805
	2.261%	30.0012	2.33%	30.0011	2.39%	30.0012	1.99%	30.0012
	2.398%	29.9015	2.39%	29.9493	2.52%	29.9602	2.09%	29.9302

OAS (%)	2.2602		2.3302		2.4012		—	
*D*_eff_	1.88		1.6012		1.3952		2.49863	
*C*_eff_	53.7065		6.6954		−118.9674		138.8735	

## Data Availability

No data were used to support this study.
